# Atypical presentation of hepatocellular carcinoma: a mass on the left thoracic wall

**DOI:** 10.1186/1471-2407-4-89

**Published:** 2004-12-01

**Authors:** Şahin Çoban, Osman Yüksel, Seyfettin Köklü, Koray Ceyhan, Meltem Baykara, Abdulkadir Dökmeci

**Affiliations:** 1 Department of Gastroenterology, Ankara University Medical School, 06100, Ankara, Turkey; 2 Department of Gastroenterology, Türkiye Yüksek İhtisas Hospital, 06100, Ankara, Turkey; 3 Division of Clinical Cytology, Department of Pathology, Ankara University Medical School, 06100, Ankara, Turkey

**Keywords:** hepatocellular carcinoma, metastases, chest wall, axilla

## Abstract

**Background:**

Hepatocellular carcinoma is a common malignancy for which chronic hepatitis B infection has been defined as the most common etiologic factor. The most frequent metastatic sites are the lung, bone, lymphatics, and brain, respectively. Metastases to the chest wall have been reported only rarely.

**Case presentation:**

We report a patient with hepatocellular carcinoma who presented with an isolated metastatic mass on the left anterolateral chest wall in the axillary region.

**Conclusions:**

Metastasis of HCC should be included in the differential diagnosis of rapidly growing lesions in unusual localizations, particularly in patients with chronic liver disease even if a primary tumor can not be radiologically identified.

## Background

Hepatocellular carcinoma (HCC) is the most frequent primary malignant tumor of the liver [1]. It is usually seen in the 6^th ^and 7^th ^decades of life, and the most common etiologic factor is chronic viral hepatitis, particularly in the presence of cirrhosis. Chronic hepatitis B has been described as the most frequent cause [2-4]. Hematogeneous extrahepatic metastases are common, with lungs, regional lymph nodes, kidneys, bone marrow and adrenals being the most frequent sites [3,4]. Rarely HCC may present as a mass without a primary liver tumor being identified [5-7].

This report presents an unusual metastasis of HCC which presented as a mass on the left chest wall in the axillary region.

## Case presentation

A 71-year-old male was admitted to our clinic with complaints of left shoulder pain, swelling in the left anterolateral chest wall, jaundice, weight loss, dyspnea and weakness. At three months prior to admission, he had noticed a less movable mass approximately 2 cm in diameter and then the tumor had grown rapidly, becoming increasingly painful during exertion. There was no history of expectoration, fever, or night sweats. The patient's physical examination revealed yellow discoloration of the sclerae, pitting edema up to the knees and a 5 cm × 6 cm fixed mass in the left axillary region. There was mild splenomegaly but no hepatomegaly, liver masses or ascites. Laboratory findings were as follows: sedimentation rate: 90 mm/hr; Hb: 11.5 mg/dL; WBC: 3900/mm^3^; Plt: 126 000/mm^3^; PT: 15.6 sec; INR:1.3; total protein: 6.5 g/dL (N: 6.4–8.5 g/dL); albumin: 2.2 g/dL (N: 3.4–4.8 g/dL); AST: 124 iu/L (N: 15–40); ALT: 52 iu/L (N: 10–40); ALP: 173 iu/L (N: 37–147); GGT: 213 iu/L (N:0–40); total bilirubin: 3.8 mg/dL (N: 0.1–1.2), and direct bilirubin: 1.57 mg/dL (N: 0–0.3). Hepatitis B virus surface antigen, IgG antibody to the core antigen, anti-HBe and HBV DNA with polymerase chain reaction were positive. HBe antigen, anti-Delta and serological markers of hepatitis C were negative.

**Figure 1:**
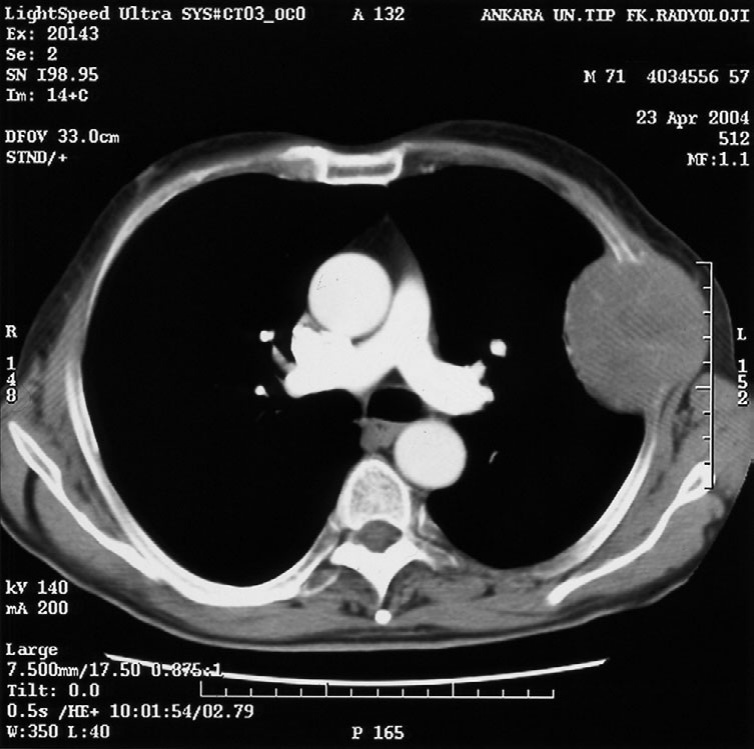
Thoracic computerized tomography showing a lesion of 7 cm × 6.5 cm in diameter on the left anterolateral chest wall in the left axillary region at the level of 4.–5. ribs with minimal compressive atelectasis and destruction of the ribs.

**Figure 2:**
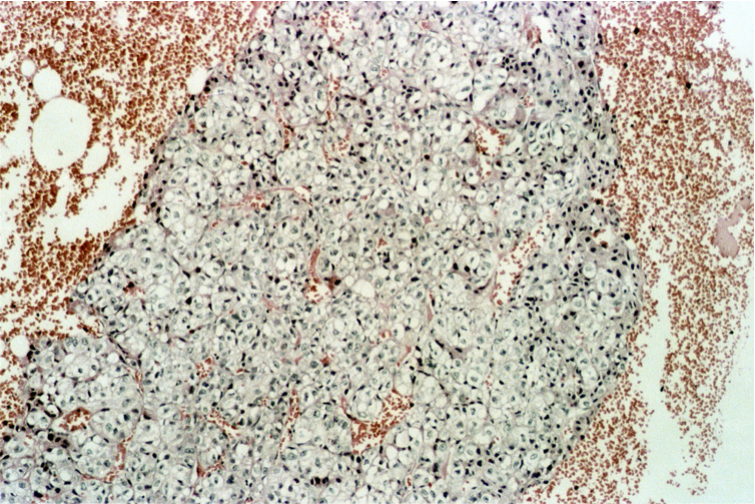
The cell block section of the axillary mass is composed of atypical hepatocytes forming thickened trabeculae and solid nests lined by endothelial cells (H& E, ×100).

Abdominal ultrasonography showed ascites, splenomegaly and diffusely nodular and heterogeneous echogenic patterns in the liver. There was no history of chronic liver disease. Upper gastrointestinal endoscopy was normal except for esophageal varices. Computerized tomography of the thorax revealed a mass on the left anterolateral chest wall (Figure 1). Cytological examination of a fine needle aspirate taken from the mass was consistent with metastatic hepatocellular carcinoma (Figure 2). Abdominal computerized tomography detected thrombosis in the right portal vein. The liver parenchyma was diffusely heterogeneous with irregular borders and without a clear mass or infiltrating lesion. There was no lymphadenopathy on thoracic, abdominal or pelvic computerized tomography. The patient had an elevated serum alpha-feto protein (AFP) level of 60 000 ng/mL (0 – 13.6 ng/ml). Cytological examination of the liver confirmed the diagnosis of hepatocellular carcinoma. The patient was discharged on palliative treatment, and he died 21 days later.

## Discussion

Although extrahepatic metastasis of HCC was reported in 18% of untreated patients in a retrospective study, metastatic lesions were found at a higher incidence in an autopsy study of deaths related to primary liver cancer [8,9]. The most common sites of extrahepatic involvement are the lungs, lymph nodes, adrenal glands, and bones [3,8,10-12]. Metastasis of HCC occurs frequently by way of intrahepatic blood vessels, lymphatic permeation, or direct infiltration. Hematogenous spread occurs with the involvement of hepatic or portal veins or the vena cava. Metastases have also been found in collaterals and varices, and this appears to have been the route of metastasis in the patient reported here. His right portal vein was thrombosed, most likely due to tumoral infiltration. Tumor cells might have passed through the left thoracic wall via portosystemic collaterals, the azygous system and finally intercostal veins. Another possible route is through subcutaneous collaterals communicating to thoracoepigastric veins and draining into the axillary vein.

Although bone metastases are seen in 10% of HCC patients [13], it infrequently appears as the first manifestation of HCC. The most common sites are the vertebra and pelvis [14]. Isolated metastases of HCC to the ribs have been rarely reported [5,6,15-17].

The establishment of the diagnosis of metastatic HCC can occasionally be problematic, particularly when the primary tumor has not been identified. To the best of our knowledge there are only two reports in the literature describing cases of solitary metastasis to the chest wall from an unknown primary HCC. One patient had an HCC metastasis involving the sternum, and the other patient had a metastasis on the right 4^th ^rib [5,6].

In the present patient, the etiology of HCC was due to chronic hepatitis B, and the HCC was diffuse in nature. Yuki et al. reported an association between HBs antigen positivity and diffuse-type HCC. Intrahepatic, hematogenous and lymphogenous metastases have been frequently observed in diffuse-type HCC [18]. Nevertheless, diffuse-type HCC is often difficult to detect on imaging studies because of its permeative appearance and heterogeneity of background chronic liver disease [19]. In our patient, although HCC was of the diffuse type, metastasis involvement was isolated. There was also no regional lymph node infiltration.

AFP, when elevated, usually correlates with tumor size. AFP doubling time is also closely related to tumor doubling time. A rapidly growing axillary mass, diffuse tumoral infiltration of the liver and a very high level of AFP in our patient are consistent with these observations. However, nearly one-fourth of patients with HCC may have normal AFP values [19].

The need for liver biopsy might be questionable in the our patient. The presence of chronic liver disease with a very high AFP level was highly suggestive for HCC. However, there are several reports in the literature describing ectopic HCC without a liver origin. These patients can be treated with surgical resection and have a good prognosis [7,20]. Furthermore, several reports have claimed that ectopic livers are particularly predisposed to developing HCC [20-22].

In conclusion, metastasis of HCC should be included in the differential diagnosis of rapidly growing lesions in unusual localizations, particularly in patients with chronic liver disease even if a primary tumor cannot be radiologically identified.

## Competing interest

The author(s) declare that they have no competing interests.

## Authors contributions

SC, involved in the patient active management and preparation of the figures. OY, carried out the literature search. SK, revised it for scientific content. KC, performed the cytological examination. MB, involved in the patient active management. AD, edited the manuscript and coordinated the submission. All authors contributed to the preparation of the manuscript. All authors read and approved the final version of the manuscript.

## Pre-publication history

The pre-publication history for this paper can be accessed here:
